# Curcumin as a permeability enhancer enhanced the antihyperlipidemic activity of dietary green tea extract

**DOI:** 10.1186/s12906-019-2545-1

**Published:** 2019-06-13

**Authors:** Ashlesha P. Pandit, Shreyas R. Joshi, Preeti S. Dalal, Vinita C. Patole

**Affiliations:** 1 Department of Pharmaceutics, JSPM’s Rajarshi Shahu College of Pharmacy and Research, Tathawade, Pune, Maharashtra 411033 India; 2 Department of Pharmaceutical Chemistry, JSPM Rajarshi Shahu College of Pharmacy and Research, Tathawade, Pune, Maharashtra 411033 India

**Keywords:** Green tea extract powder, Epigallocatechin-3-gallate (EGCG), *Camellia sinensis*, Hyperlipidemia, Tea bags, Curcumin

## Abstract

**Background:**

Green tea has polyphenols like flavonoids and catechins; mainly epigallocatechin-3-gallate (EGCG), epicatechin gallate (ECG), epigallocatechin (EGC) and epicatechin (EC), out of which EGCG is of higher abundance. EGCG has shown preventive role in hypercholesterolemia. However, due to low oral bioavailability, a need arises to improve its membrane permeability and transporter-mediated intestinal efflux. Therefore, an attempt was made to enhance permeability and bioavailability of EGCG using curcumin to treat hyperlipidemia. Further, it was formulated in herbal tea bags to achieve patient compliance.

**Methods:**

EGCG extracted from green tea leaves was confirmed by High Performance Thin Layer Chromatography. Green tea extract (GTE), curcumin and their mixtures were subjected to Fourier Transform Infra-Red spectroscopy and Differential Scanning Calorimetry for compatibility studies. Powder formulation was prepared comprising GTE, curcumin, sucralose and cardamom.

**Results:**

Ex-vivo study was performed on everted goat intestine, analyzed by HPLC and demonstrated highest permeation of GTE:curcumin (220:50) (53.15%) than GTE (20.57%). Antihyperlipidemic activity was performed in rats for 15 days. Blood sample analysis of rats of test groups (formulation and GTE solution) fed on high fat diet showed (mg/dl):cholesterol 80 and 90, triglycerides 73.25 and 85.5, HDL 50.75 and 46, LDL 43.9 and 46, VLDL 14.65 and 17.1 respectively with significant lipid regulating effect.

**Conclusion:**

Curcumin enhanced permeability of EGCG. Therefore, P-glycoprotein pump inside intestine can be potential mechanism to enhance permeability of EGCG. Thus, EGCG-curcumin herbal tea bag is promising nutraceutical to treat hyperlipidemia in day-to-day life achieving patient compliance.

## Background

Herbal-based drugs are reported to have curative value for various disorders in traditional medicines [1]. Chronic diseases can be prevented by certain food possessing medicinal functions. Polyphenols are naturally occurring compounds found largely in the fruits, vegetables, cereals and beverages. Use of polyphenols has been observed to have their passable beneficial effects on human health [2]. Tea is one of the most widely consumed beverages, especially in Asia, for its long-purported health benefits [3]. Due to convincing evidence that tea is a cup of life, its consumption has increased substantially worldwide. Green tea is a variety of tea brewed from the dried leaves of the *Camellia sinensis* plant, belonging to Family *Theaceae*. Unlike other teas, green tea is not fermented. Green tea production and manufacture has spread in many countries in Asia. Green tea contains polyphenols like flavonoids containing catechins; mainly epigallocatechin-3-gallate (EGCG), epicatechin gallate (ECG), epigallocatechin (EGC), and epicatechin (EC), out of which EGCG is of higher abundance [4]. Due to oxidation of the chemical constituents of tea leaves, black tea lose its beneficial effects. However, green tea leaves have an antioxidant property [5].

Dyslipidemia is the condition mainly characterised by abnormal levels of cholesterol, triglycerides and lipoproteins, occurring more frequent in obese people. Dyslipidemia may lead to atherosclerosis induced cardiovascular complications such as high blood pressure, cardiac dysfunction, ischemic heart disease and coronary artery disease [6]. Concentration of total cholesterol in serum reflects different concentrations of lipoproteins in the serum. The major classes of lipoproteins are chylomicrons, very low density lipoproteins (VLDL), low density lipoproteins (LDL) and high density lipoproteins (HDL). In hyperlipidemia, the levels of cholesterol, triglycerides, LDL and VLDL are increased while level of HDL is decreased. Modern lifestyle and food habits are mainly responsible for this condition, and thus can be treated by diet based therapy. Green tea catechins play preventive role in obesity and related disorders mainly hypercholesterolemia and hyperglycaemia. Chronic treatment with EGCG can modulate dyslipidemia. Green tea extract has more lipidemia modulating effects than black tea extract [7].

The major components of green tea are catechins. The major catechins comprise epigallocatechin-3-gallate (EGCG), epicatechin gallate (ECG), epicatechin (EC), and epigallocatechin (EgC). Most studies have attributed the various beneficial effects of green tea to EGCG, the major constituent in green tea [8]. EGCG, a type of catechin, is an ester of epigallocatechin and gallic acid. The most abundant catechin in tea is polyphenol. A great deal of attention is received by this dietary polyphenolic compounds as they are known to restore the balance between the natural antioxidants and free radicals by direct scavenging of reactive oxygen species and by enhancing the activity of natural antioxidant enzymes [9]. EGCG is water soluble and is not greatly influenced by high temperature conditions such as boiling water. It binds to many biological molecules and influences the activity of various enzymes. EGCG blocks carcinogenesis by affecting a wide array of signal transduction pathways including Jun NH_2_-terminal kinase/ signal transducers and activators of transcription, mitogen-activated protein kinase, phosphatidylinositol- 3-kinase/AKT [10, 11]. EGCG has shown many health promoting effects such as hypoglycaemic, hypolipidemic, anti-cancer, cardioprotective and anti-infective activity [12]. EGCG is reported to act by reducing fat absorption from gut [13] (as well as by directly inhibiting HMG Co-A reductase enzyme in hyperlipidemia [14]. Despite of these promising uses of green tea, oral bioavailability of EGCG is very low [11].The absolute bioavailability of EGCG after oral administration in rats is found to be 0.1% [15]**.** Low bioavailability of EGCG seems to be related to its poor membrane permeability and transporter-mediated intestinal efflux [16]. Therefore, a need arises to enhance oral bioavailability of EGCG.

Curcumin, obtained from *Curcuma longa*, is used as bio-enhancer for anti-microbial agents and anti-cancer drugs. It shows strong antioxidant, anti-inflammatory, anti-mutagenic and anti-carcinogenic properties. Curcumin can recover chemically-induced oxidative stress as well as increase xenobiotic detoxifying enzymes’ activities in both the liver and kidneys and suppress lipid peroxidation [17]. Curcumin has low solubility and low permeability from intestine due to P-glycoprotein pump (P-gp) [18]. Curcumin acts by two mechanisms:suppression of drug metabolising enzymes in liver and inducing changes in drug transporter P-gp. Curcumin is initially absorbed from intestine, but is effluxed again inside intestine by P-gp. Curcumin and EGCG both are effluxed by P-gp; hence curcumin can play a pivotal role of inhibiting P-gp, thereby enhancing permeation of EGCG by inhibiting its efflux [19].

Piperine was found to enhance bioavailability of EGCG [20]. Encapsulation of catechin and epigallocatechin gallate (EGCG) in chitosan nanoparticles enhanced their intestinal absorption [21]. EGCG microparticles prepared by using Eudragit S100 enhancedits bioavailability by increasing contact time between nanoparticles and gut [22].

An attempt was made to use innovative method to enhance permeability of green tea constituents to improve antihyperlipidemic activity using curcumin, by inhibiting its efflux inside intestine. EGCG obtained from green tea extract was formulated in herbal tea bags with an advantage of delivering formulation in liquid form to achieve patient compliance.

## Materials and methods

### Materials

Curcumin was procured from Loba Chemie, India. Green tea leaves and cardamom powder were procured from Ayurvedic Centre, Pune, India. All other chemicals were of analytical grade and were used as received.

### Methods

#### Extraction of green tea leaves

Green tea leaves (200 g) reduced to suitable size were brewed in 2000 ml of distilled water at 50 °C for 10 min in a thermostat heating mantle (J B Scientific, India). Temperature was increased to 80 °C and heated for further 10 min. Solution obtained was dried to powder using vacuum evaporator (Equitron-Evator Medica Instruments, Mumbai).Powder was triturated to reduce particle size for further studies [23].

### Characterizations of green tea extract powder

#### Yield and flow properties

Green tea extract powder (GTE) was weighed and evaluated for properties such as physical appearance, density, compressibility index, Hausner’s ratio and angle of repose. Preliminary phytochemical screening was done for EGCG by performing catecholic tannin test as follows: 0.5 ml of extract was dissolved in 1 ml of water and mixed thoroughly. Two drops of ferric chloride solution was added to it. Solution was observed for green black colour for presence of catecholic tannin.

### Analysis of green tea extract by HPTLC for EGCG

Green tea extract was characterized by HPTLC (CAMAG, Switzerland). The mobile phase used was toluene: acetone: formic acid (9:9:2).Sample solution of 1 to 15 μL was applied as 8 mm band from lower edge of the plate. Plates were developed over 6 cm from lower edge of plate using an unsaturated trough chamber [24, 25].

Derivatization for polyphenols was done by heating the plate at 100 °C for 2 min followed by dipping the hot plate in fast blue salt B reagent. Further, plate was dried in a fume hood for 5 min after derivatization and evaluated for R_f_ value.

### Infra-red spectroscopy

Curcumin, green tea extract and mixture were analysed by FT-IR (Alpha Bruker, Germany) to get spectra. Samples in powder form were placed on sample holder in sufficient amount and tightened with upper probe. Samples were analysed using Opus software to get IR spectra.IR spectrum of green tea extract was compared with standard spectrum to confirm EGCG.

### Differential scanning calorimetry

Green tea extract, curcumin and their mixture (1:1) were identified using differential scanning calorimetry (DSC1, Mettler Toledo, Switzerland).

### Determination of tensile strength of tea bags

Tensile strength of tea bags was measured using CT3 Texture Analyzer (Brookfield Engineering Corporation, USA) intension mode by using TA-DGA (Dual Grip Assembly) accessory in TA-P-KIT.

### Formulation of tea bags

#### Preliminary trials

Preliminary study of formulation was performed by trial and error batches with varying concentrations of green tea extract (150 to 300 mg) and curcumin powder (25 to 100 mg). Ex-vivo study of these batches was performed to study effect of curcumin on permeability of green tea extract. Batches showing good permeability were selected for further formulation.

Green tea extract (180 to 220 mg) and curcumin powder (25 to 75 mg) were lightly triturated in mortar and pestle. Sucralose and cardamom powder were added as artificial sweetener and flavouring agent respectively, to the above mixture, again triturated and mixed in planetary mixer (Avon, India) to obtain tea powder mixture. Formulation batches of tea powder (F1 to F9) are as shown in Table 1. A single use tea powder (about 2.2 g) was filled carefully in tea bags and sealed. Tea solution was prepared by dunking tea bag and moving up-down for 5 min in 100 ml preheated (80 °C) distilled water. Solution was allowed to cool at 37 °C and used for further studies.

**Table 1:** Formulation of green tea extract in tea bags

Ingredient	F1	F2	F3	F4	F5	F6	F7	F8	F9
Green tea extract (mg)	220	220	220	200	200	200	180	180	180
Curcumin (mg)	25	50	75	25	50	75	25	50	75
Sucralose (mg)	2000	2000	2000	2000	2000	2000	2000	2000	2000
Cardamom (mg)	q. s.	q. s.	q. s.	q. s.	q. s.	q. s.	q. s.	q. s.	q. s.

### Evaluation of tea bags

#### Drug content

Drug content (EGCG) of F1 to F9 batches was determined. Tea bags were dunk in 100 ml water (pre-heated to 80 °C), moving up-down for 5 min and cooled to 37 °C. Aliquot (5 ml) was withdrawn after 5 min. Suitable dilutions were made and drug content was estimated at 274 nm using UV spectrophotometer (1800, Shimadzu, Japan) and distilled water as a blank solution.

### Ex-vivo study

**Fig. 1:**
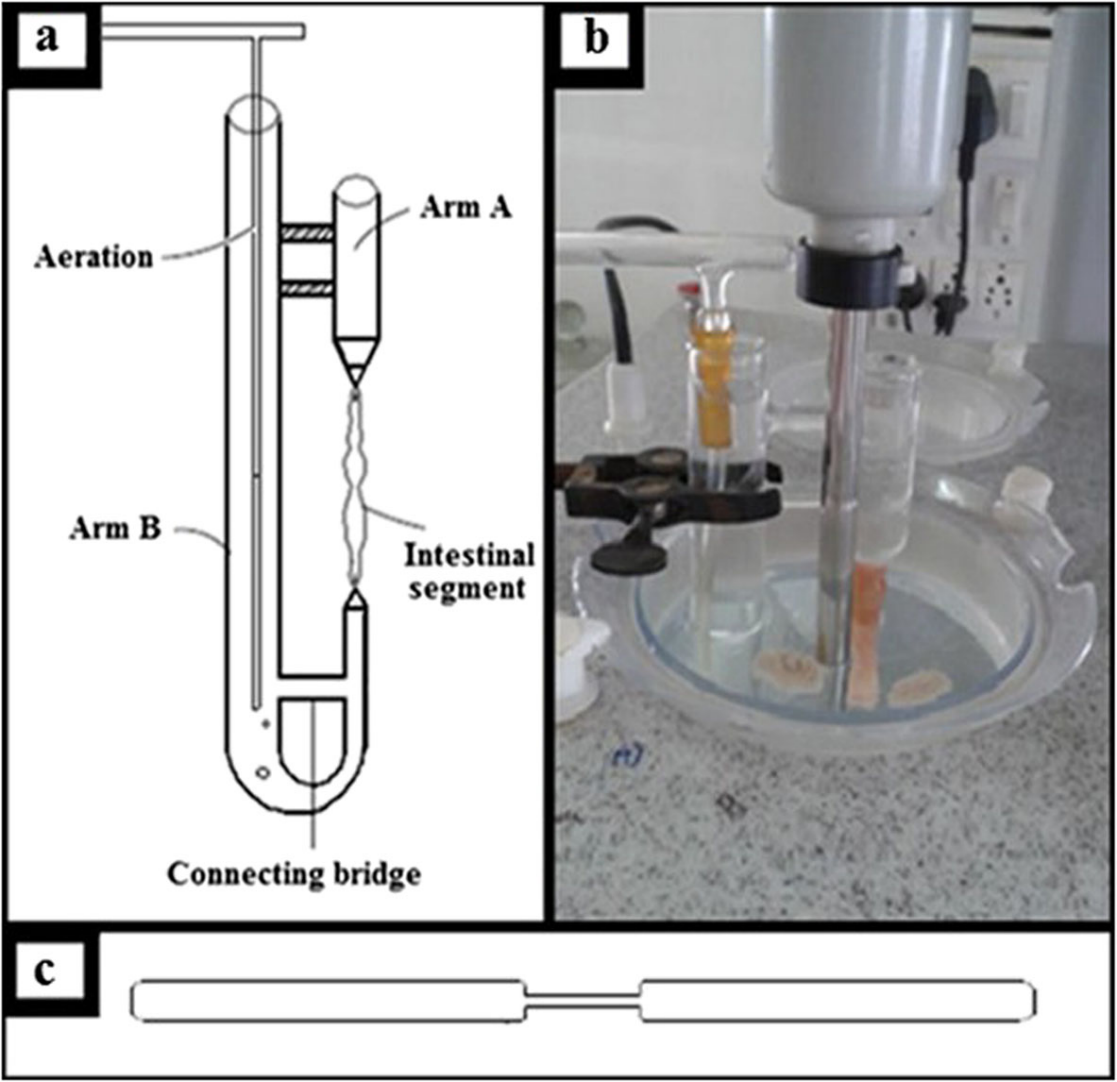
Everted gut sac apparatus **a** U-shaped apparatus, **b** Assembly, **c** Glass rod

In vitro continuous dissolution–absorption system design was used to study amount of drug permeated intestine using everted intestine [26]. Fresh small intestine of goat was brought from slaughter-house with supplement of tyrode solution pH 6.5 (8 g NaCl, 1 g Glucose, 1 g NaHCO_3_, 0.2 g CaCl_2_, 0.2 g KCl, 0.1 g MgCl_2_, 0.05 g NaH_2_PO_4_). Intestine was carefully manoeuvred to find ileum part. Suitable length of ileum (5 cm) was cut off, everted using glass rod (Fig. 1c), cleaned in supplement of fresh tyrode solution and attached to U-shaped apparatus (Fig. 1a). In-vitro drug permeation study of formulation was carried out in1000 ml container having phosphate buffer pH 6.8 (800 ml) as dissolution medium. To this, an ex vivo U-tube apparatus containing same buffer solution was assembled (Fig. 1b). Temperature was maintained at 37 ± 0.5 °C throughout study.

GTE and formulations (F1 to F9) (100 ml) were studied using the above mentioned assembly (Fig. 1b). Formulation was added in the dissolution medium and allowed to diffuse through an everted intestine into an ex-vivo apparatus. Five ml of aliquot was withdrawn from arm B (Fig. 1a) after every 15 min time interval for 150 min, replacing equal volume of the solution and maintaining the temperature [27]. Drug was diffused from dissolution medium (mucosal side) to the serosal side (absorption compartment). The amount of diffused drug was analyzed UV spectrophotometrically. The experiment was repeated thrice using fresh dissolution medium as well as fresh intestinal segment each time.

### Selection of best formulation

Curcumin was selected as permeation enhancer. The criterion for selection of the best batch was the formulation showing highest permeation of green tea extract through intestine. The best batch was subjected to UV-spectroscopy and RP-HPLC for analysis.

### HPLC analysis of formulation

The best batch obtained at ex-vivo study was subjected to reverse phase HPLC using HPLC system (Jasco, PU-2080Plus pump, UV-2075Plus detector) with C-18 column (250 mm × 40 mm) having 5 μ particle size at 274 nm. Mobile phase used was acetonitrile: 0.1% ortho phosphoric acid (10:90). Flow rate was 1.2 ml/min. Calibration curve was performed with standard concentrations of 10 ppm to 70 ppm with run time of 20 min [28].

### Diffusion study of formulation through tea bags

Amount of green tea extract diffused through tea bags was determined by dunking tea bag (220 mg) into a beaker with preheated (80 °C) distilled water (100 ml) on magnetic stirrer (J. B. Scientifics, India) to simulate dip-dip movement of tea bag formulation. At predetermined time interval of 1 min, one ml of aliquot was withdrawn, replacing same volume with distilled water and maintaining same temperature. After suitable dilution, samples were analysed by spectrophotometric method.

### In-vivo study

Healthy Sprague Dawley rats (10 male and 10 female, weighing between 200 and 250 g and of age 6–7 weeks) were used to study hyperlipidemic activity. Animals were housed at temperature 21 ± 1 °C and humidity 45–55% in polycarbonate cages by exposing alternate 12 h light and dark cycle.

The rats were divided into 5 groups (Group I to V), containing 4 rats in each group, two male and two female: Group I: Control; Group II: Negative control; Group III: Standard (Marketed group A); Group IV: Test group with high fat diet ad libitum and GTE solution (Test group B); Group V: Test group with high fat diet ad libitum and GTE plus curcumin solution (F2) (Test group C).

Initially the rats were acclimatized to laboratory conditions and were fed with commercially available standard diet and water. After initiation of the study, control group (Group I) was fed with normal diet and water ad libitum. Negative control group (Group II) was fed with high fat diet ad libitum(casein 12%, corn starch 42.96%, soybean oil 25%, cholesterol 1%, coline 0.04%, salt mixture 5%, vitamin mixture 1% and cellulose 13%). Test group A (Group III) was given high fat diet ad libitum for 15 days and liquid solution of marketed preparation atorvastatin (10 mg once a day) for further 15 days. Test group B (Group IV) was given high fat diet ad libitum for 15 days and GTE in solution form (0.5 ml), once a day for further 15 days with a dose of 100 mg/kg/day. Test group C (Group V) was given high fat diet ad libitum for 15 days and F2 formulation in solution form (0.5 ml), once a day, for further 15 days with a dose of 100 mg/kg/day. At the end of study period, all the animals were anesthetized using isoflurane anaesthesia, blood samples of all groups were collected from retro-orbital sinuses and analysed for serum triglycerides, cholesterol, low density lipoproteins (LDL), High Density Lipoproteins (HDL), and Very Low density lipoproteins (VLDL) [29–31]. After collection of samples, the animals were humanly euthanized/ sacrificed using carbon-dioxide asphyxiation. The animal study was carried out as per the protocol approved by the Institutional Animal Ethics Committee of PRADO (Preclinical Research and Development Organisation) India, constituted as per the guidelines laid down by Committee for Purpose of Control and Supervision of Experiments on Animals (CPCSEA). The protocol approval no. is IAEC- 16-001.

### Statistical analysis

Statistical analysis was performed by applying Graphpad Prism version 5 software (GraphPad Software Inc., La Jolla, CA). The experimental results were expressed as the mean ± standard error of mean. The results were analyzed using one-way analysis of variance (ANOVA) with Bonferroni post-tests, where *p* < 0.001 was considered as statistically significant.

### Stability study

Herbal products are prone to microbial growth. Hence, stability of these formulations should be studied. Stability study of best batch was carried out to get a stable product which assured safety and efficacy, till shelf life, at defined storage and package conditions. Stability studies were carried out as per ICH guidelines to assess the combined effect of drug and excipients on stability of formulation. Best formulation was packed in tea bags, further in cardboard carton and kept in stability chamber (Thermolab, India) at 40 °C ± 2 °C/ 75%RH ± 5%RH. Samples were evaluated for any change in colour of powder, odour, microbial growth and drug content at 15 days interval for three months. Ex-vivo study was performed at the end of three months.

## Results

### Characterisation of green tea extract

Yield of green tea extract was found to be 7.45%. The dried and triturated form of powder extract was brownish in colour, having pungent odour and yield of 10%.

### Flow properties

Angle of repose of GTE powder was found to be 43°, which demonstrated passable flow character. Compressibility index and Hausner’s ratio was 20% and 1.25 respectively, which indicates good flow characteristic.Preliminary phytochemical screening done for EGCG by performing catecholic tannin test showed green black colour and confirmed the presence of catecholic tannin.

### Analysis of EGCG in green tea extract by HPTLC

HPTLC of green tea extract was carried out to confirm the presence of active molecule mainly epigallocatechin gallate in the green tea extract. HPTLC was performed keeping experimental conditions constant. Sample plate on which green tea extract was applied showed strong peak at R_f_ value 0.12.Standard R_f_ value for pure EGCG was found to be 0.12. The obtained results were in agreement with earlier work [24, 25].

### Infra-red spectroscopy

**Fig. 2:**
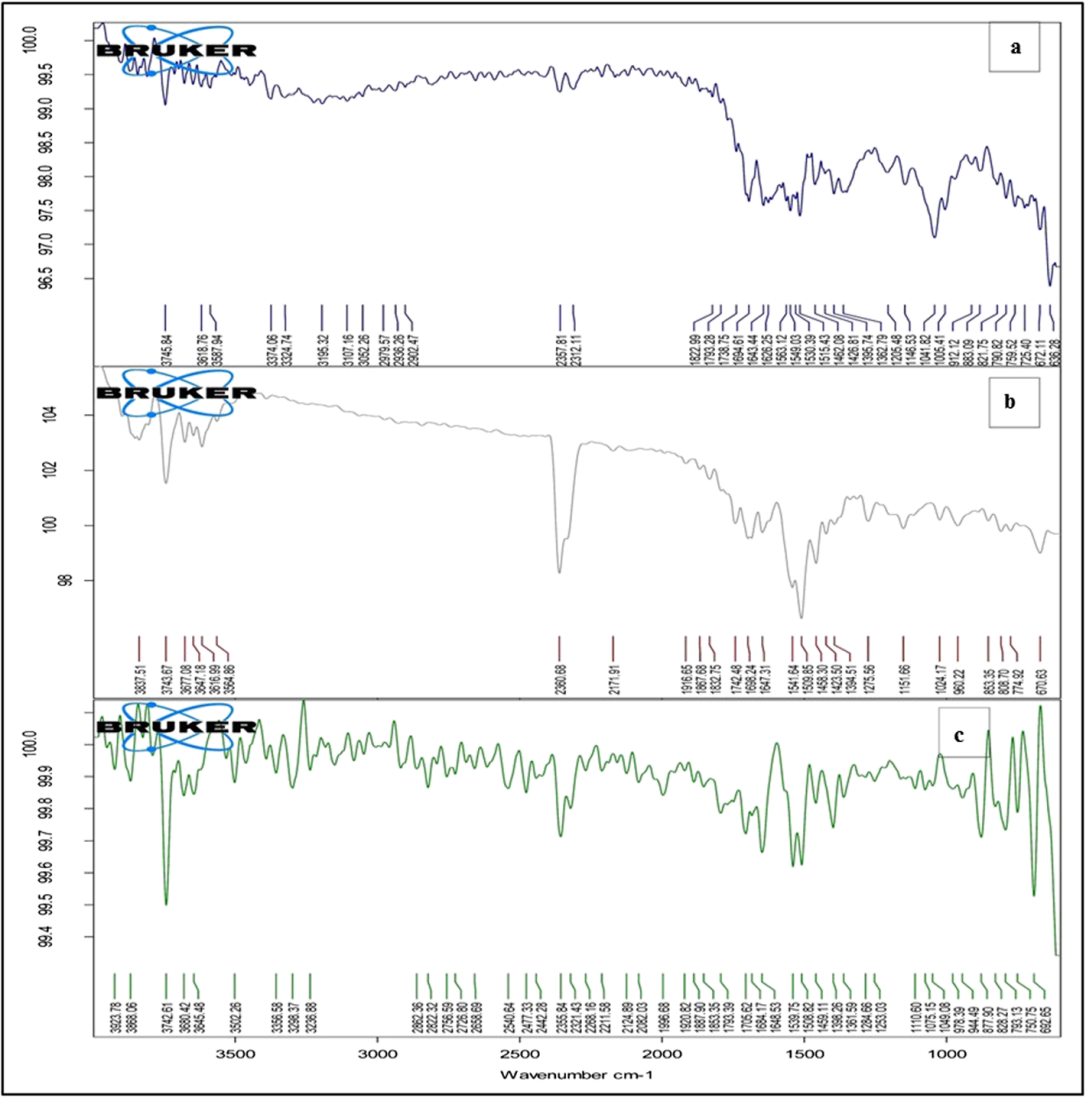
Infrared spectra of **a** Green tea extract, **b** Curcumin, and **c** Mixture

IR spectrum of green tea extract (Fig. 2a) showed sharp peak at 3745 cm^− 1^ demonstrating presence of -OH group. Strong peak at 1694 cm^− 1^ showed presence of C=O group of aryl ketone. Multiple bands at 1515 cm^− 1^, 1530 cm^− 1^, 1549 cm^− 1^, 1563 cm^− 1^ stretch indicated presence of aromatic C=C group. One band at 1738 cm^− 1^ showed presence of C=O stretch group of ester and two bands at 1041 cm^− 1^ and 1005 cm^− 1^ indicated exist of C-O stretch of ester group.

IR spectrum of pure curcumin (Fig. 2b) indicated broad strong peak at 3837 cm^− 1^ indicating presence of hydrogen bonded alcoholic –OH group. Strong sharp free peak at 3743 cm^− 1^ indicated presence of alcoholic –OH group. Peak at 1742 cm^− 1^ indicated presence of aldehyde C=O group. Multiple bands at 1509 cm^− 1^ and 1541 cm^− 1^ showed presence of aromatic C=C group. Peak at 1151 cm^−1^and 670 cm^−1^indicated alcoholic C-O stretch group and alkene = C-H group respectively.IR spectrum (Fig. 2c) of mixture of extract and curcumin showed presence of all bands of both components indicating no chemical interaction of both the components.

### Differential scanning calorimetry

**Fig. 3:**
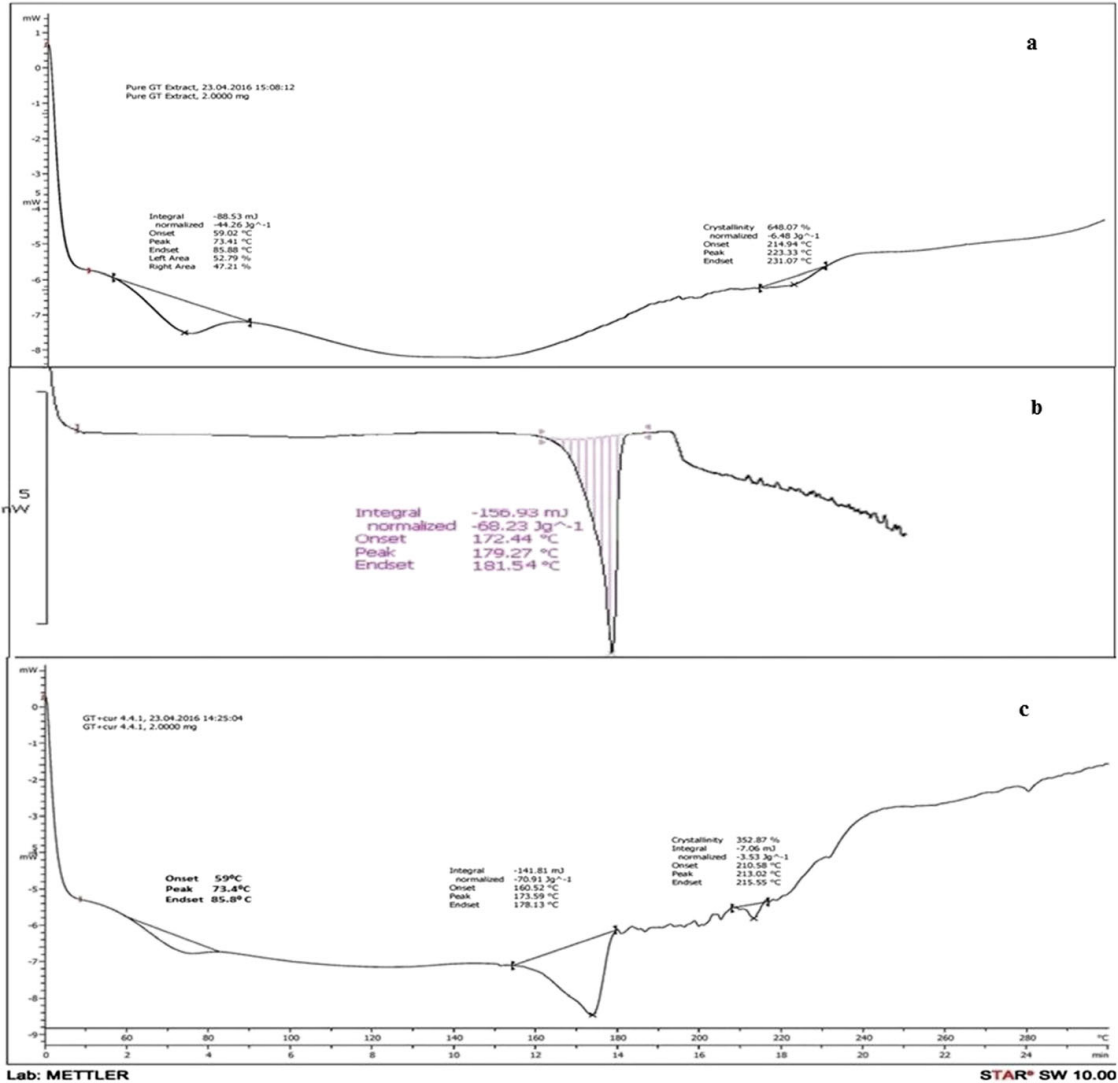
DSC thermogram of **a** Green tea extract, **b** Curcumin, **c** Mixture

Figure 3a shows thermogram of green tea extract. A broad peak was observed at 73 °C, and a small plateau at 223 °C. Figure 3b shows thermogram of pure curcumin, which indicated a sharp peak near 179 °C. Figure 3c shows thermogram of mixture of green tea extract and curcumin which demonstrated peaks due to physical interaction, indicating no chemical interaction between two components [32].

### Tensile strength of tea bag

Good strength and integrity of tea bag was obtained at peak load of 4210 g at trigger load of 5 g. The result indicates strength and integrity of tea bags to protect content from damages during transport and formulation in hot water.

### Evaluation of tea bag formulation

#### Drug content

Drug content was found as follows: F1 (97.2%), F2 (96.21%), F3 (97.4%), F4 (96.5%), F5 (95.25%), F6 (95.45%), F7 (94.25%), F8 (93.98%) and F9 (92.8%).

### Ex-vivo studies by UV spectrophotometer

**Fig. 4:**
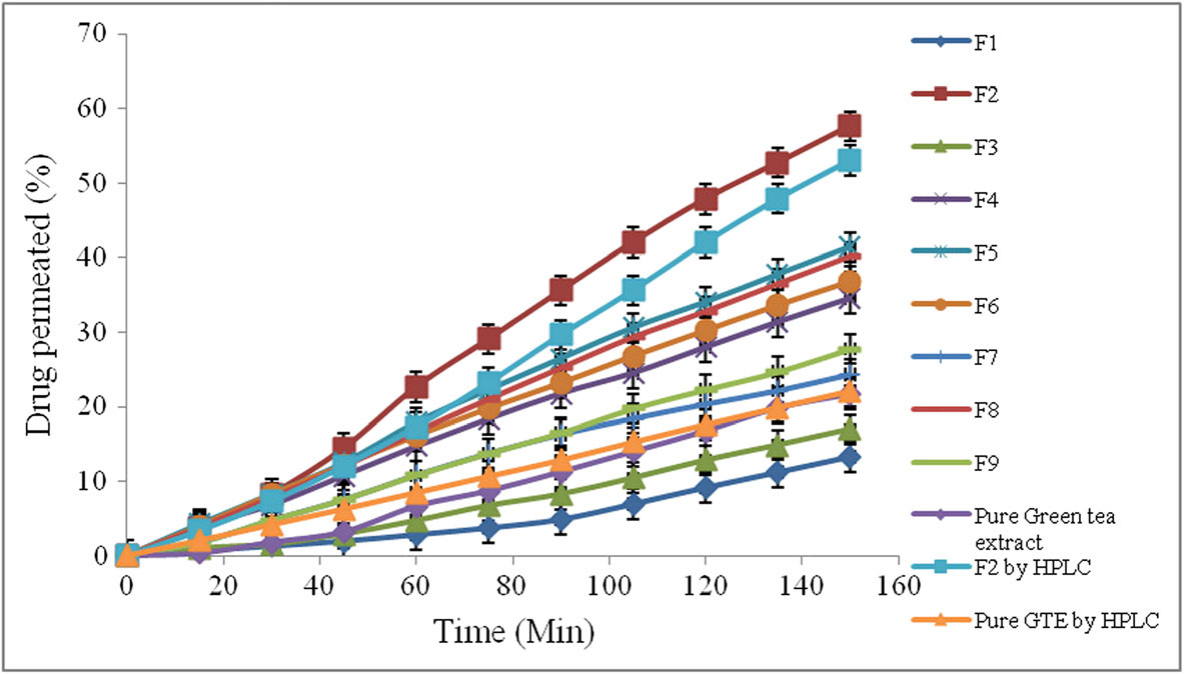
Ex-vivo study of drug permeated through intestine

Drug permeation of GTE was found to be 21.64%. From Fig. 4, it was inferred that at equal concentrations of curcumin in F4 (34.52%) and F7 (24.28%), curcumin might have permeated through intestine, as green tea extract particularly EGCG, inhibited the P-glycoprotein pump. In case of F1 (13.27%), due to insufficient quantity of curcumin, P-gp pump might not be inhibited resulting in less permeation of GTE. At highest concentrations of curcumin in F6 (36.8%) and F9 (27.73%), curcumin itself might got absorbed into the systemic circulation due to inhibition of P-gp pump by EGCG. F5 (41.39%) and F8 (40.09%) showed intermediate permeation compared to other formulations. F2 showed highest permeation (57.64%) of ECGC amongst all formulations owing to inhibition of P-gp pump by curcumin. P-glycoprotein pump inside intestine can be potential mechanism based on literature to enhance permeability of EGCG. Therefore, 50 mg and 220 mg of curcumin and GTE respectively were sufficient to inhibit P-gp pump of EGCG, enhancing the permeability of GTE.

### Ex-vivo studies by RP-HPLC

Samples of ex-vivo studies of F2 and pure GTE were analysed and confirmed by HPLC. HPLC results were in close agreement with earlier results obtained by UV-visible spectrophotometer. The drug permeated across the intestine from pure GTE and formulation (F2) was found to be 20.67 and 55.02% respectively by HPLC method.This study confirmed pivotal role of curcumin as permeation enhancer to enhance bioavailability of GTE from the intestine, by inhibition of P-gp pump and by enzyme inhibition.

### Diffusion study

Diffusion study of tea bag formulations showed greatest amount of GTE and curcumin diffused across the tea bag membrane (96 and 97% respectively in 7 min).

### In-vivo studies

**Fig. 5:**
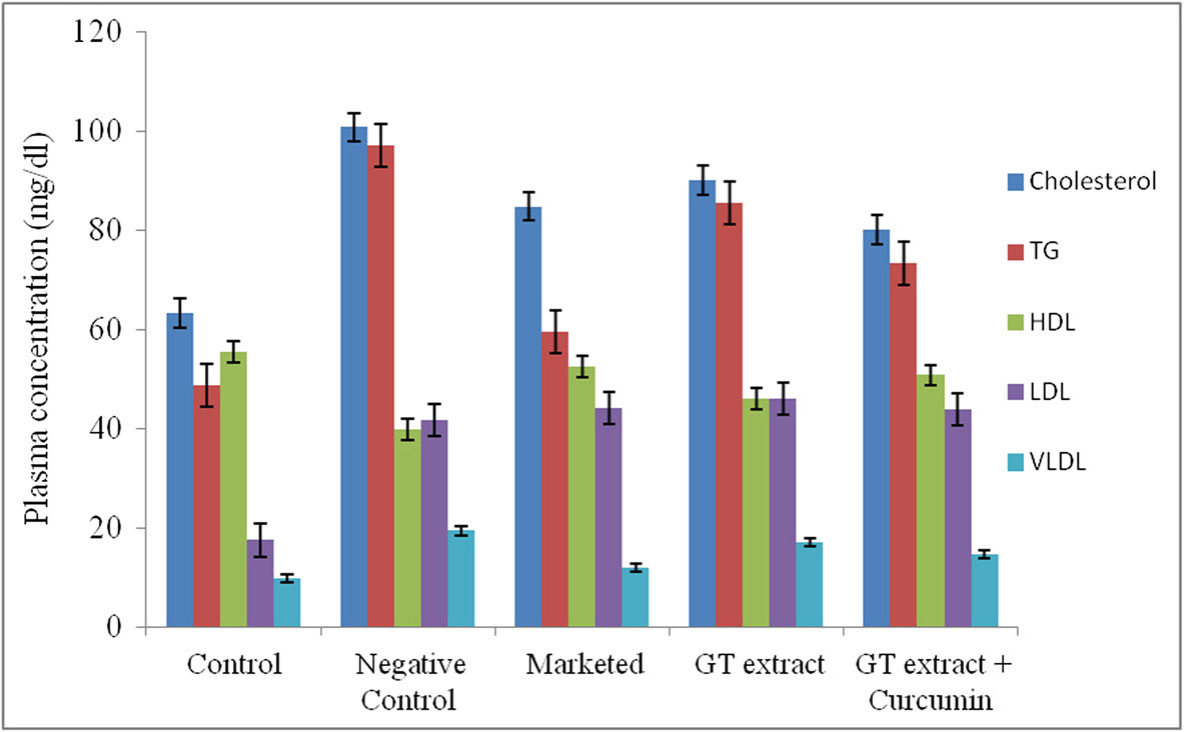
Lipid profile of in-vivo study in rats. Data is expressed as Mean ± SD of n = 4, statistical significance *P* < 0.01

Results of blood samples analysed for cholesterol, triglycerides, LDL, HDL and VLDL are shown in Fig. 5.Serum lipid content in Group II (negative control) confirmed significant induction of hyperlipidemia. Serum cholesterol level of Group II was 100.75 ± 2.5 mg/dl with *p* < 0.001. This was easily comparable with serum cholesterol level of Group I (control group) (63.25 ± 4.19 mg/dl, p < 0.001).Group III (Test A) showed regulation of lipid with lowered cholesterol (84.74 ± 4.34 mg/dl, p < 0.001), lipoproteins (LDL = 44.15 ± 2.53 mg/dl, VLDL = 11.9 ± 0.62 mg/dl with p < 0.001) and triglycerides (59.5 ± 3.1 mg/dl, p < 0.001) and increased values of HDL (52.5 ± 2.64 mg/dl). Group IV (Test B) showed significant lipid regulating effects as compared to negative control group (cholesterol = 90 ± 1.82 mg/dl, triglycerides = 85.5 ± 2.08 mg/dl, HDL = 46 ± 2.16 mg/dl, LDL = 46 ± 2.16 mg/dl, VLDL = 17.1 ± 0.41 mg/dl) with p < 0.001. Group V (Test C) (F2) showed more significant lipid regulating effects than Group IV (Test B), depicting effectiveness equal to standard marketed preparation in Test A group (cholesterol = 80 ± 1.82 mg/dl, triglycerides = 73.25 ± 2.21 mg/dl, HDL = 50.75 ± 1.7 mg/dl, LDL = 43.9 ± 1.93 mg/dl, VLDL = 14.65 ± 0.44 mg/dl with p < 0.001) [33].

### Stability study

Formulation showed no microbial growth and retention of its brownish colour. Drug content of formulation after 15 days and three months was 96.11 and 96.01% respectively. Ex-vivo study showed drug permeation of 57.23 and 57.12% respectively, after 15 days and three months. Thus, tea bag formulation was found to be stable.

## Discussion

### Phytochemical investigation

Preliminary phytochemical screening and HPTLC study confirmed exist of catecholic tannin and EGCG in green tea extract.IR spectrum and DSC thermogram of individual and mixture of extract and curcumin showed presence of all bands and peaks of both components indicating no chemical interaction of both the components. Drug content of F1 to F9 batches was found in the range of 91 to 97%, thus confirming uniformity of content from batch to batch.

### Ex-vivo studies

Ex-vivo studies confirmed highest amount of EGCG permeated across the gut, through formulation F2, analyzed by both UV and HPLC method. Therefore, F2 was selected as the best batch. Diffusion study of tea bag formulations showed maximum amount of GTE and curcumin diffused across the tea bag membrane. Thus, formulation F2 containing mixture of GTE and curcumin depicted higher lipid regulating effect than pure GTE. Reason for this could be enhanced bioavailability of GTE in presence of curcumin.

### In vivo studies

In vivo study showed no toxic effects of GTE or curcumin in rats at 100 mg/kg/day dose of formulation. Rats were healthy throughout the study. In-vivo study, thus, supported ex-vivo study of increased permeability and then bioavailability of GTE by adding curcumin, leading to more significant serum lipid regulating effect in rats. The results obtained are in close agreement with earlier observations of green tea extract and lactobacillus strain [29]. One of the underlying mechanism by which EGCG affects lipid metabolism is by interfering with the luminal emulsification, hydrolysis, and micellar solubilization of lipids and cholesterol in the digestive tract, which in turn decrease uptake of lipids and cholesterol absorption [34]. This may be due to complex formation of EGCG with lipids and lipolytic enzymes. Green tea or catechins may also influence the uptake and intracellular processing of lipids and assembly and secretion of chylomicrons [35]. EGCG with curcumin thus can be a potential candidate to reduce hyperlipidemia. Promising results can be expected with increase in time period of the study. Stability study showed stable formulation of tea bags.

## Conclusion

Formulation of herbal tea bags was developed containing green tea extract using curcumin as a permeation enhancer. Curcumin enhanced permeation of green tea extract, mainly epigallocatechin gallate (EGCG), which is the most important active constituent of green tea extract. Formulation fulfilled dose requirement and regulated serum lipid levels significantly compared to pure green tea extract (without curcumin), with no side effects. Curcumin, thus can be an effective permeation enhancer to increase bioavailability of EGCG present in green tea, effectively reducing the hyperlipidemia. Ease and convenience of tea bags make it suitable for even bed-ridden patients. The novel concept of tea bags achieves the patient compliance.

## Data Availability

All data generated or analyzed during this study are included in this published article.
